# Serum magnesium predictive value in hepatocellular carcinoma patients on first-line immunotherapy: a retrospective study

**DOI:** 10.3389/fimmu.2025.1732557

**Published:** 2026-01-05

**Authors:** Xi Cheng, Wei Wang, Ya-nan Xue, Hong-lin Tang, Da Li

**Affiliations:** Department of Medical Oncology, Sir Run Run Shaw Hospital, Zhejiang University School of Medicine, Hangzhou, China

**Keywords:** clinical outcome, hepatocellular carcinoma, immune checkpoint inhibitor, predictive value, serum magnesium

## Abstract

**Background:**

Serum magnesium plays a critical role in modulating immune responses and enhancing anti-tumor immunity in cancer patients. However, its predictive significance in relation to progression-free survival (PFS) and disease control rate (DCR) among hepatocellular carcinoma (HCC) patients undergoing first-line immunotherapy remains uninvestigated.

**Methods:**

This retrospective study analyzed HCC patients treated with immune checkpoint inhibitors (ICIs) in a derivation cohort and an independent validation cohort. Patients were enrolled based on predefined inclusion criteria. The primary endpoint was to assess the association between baseline serum magnesium levels prior to immunotherapy initiation and the disease control rate. The optimal cut-off value of serum magnesium for predicting disease control was determined using receiver operating characteristic (ROC) curve analysis. PFS was evaluated using the Kaplan–Meier method.

**Results:**

A total of 110 hepatocellular carcinoma patients treated with ICIs were enrolled in the derivation cohort. The DCR was 78.18%. ROC analysis identified a baseline serum magnesium level ≥0.785 mmol/L as predictive of disease control, with a sensitivity of 89.4% and specificity of 87.5% (AUC 0.869). Patients with baseline Mg^2+^ ≥0.785mmol/L demonstrated significantly longer median PFS compared to those with baseline Mg^2+^<0.785mmol/L (12.83 months *vs*. 3.45 months, P< 0.001, hazard ratio: 0.269, 95% confidence interval: 0.165–0.438). The DCR was 30% in the low-Mg^2+^ group and 96.25% in the high-Mg^2+^ group. Subgroup analyses revealed that patients with macrovascular invasion or extrahepatic metastasis, as well as those without macrovascular invasion or extrahepatic metastases in the high-Mg^2+^ group exhibited significantly longer median PFS compared to those in the low-Mg^2+^ group. Both univariate and multivariate analyses confirmed that serum magnesium level is an independent predictive factor of PFS for patients receiving immunotherapy. In the validation cohort (n=48), patients with Mg^2+^ ≥0.785 mmol/L showed significantly longer median PFS (17.13 months *vs*. 5.20 months, P = 0.037, hazard ratio: 0.304, 95% confidence interval: 0.131–0.701) and higher DCR compared to those with Mg^2+^<0.785mmol/L.

**Conclusion:**

Elevated serum magnesium levels prior to first-line immunotherapy are associated with improved clinical outcomes in HCC. Serum magnesium demonstrates significant predictive value and could serve as a cost-effective, accessible biomarker for guiding immunotherapy strategies in HCC patients.

## 1 Introduction

Liver cancer constitutes the sixth most common cause of cancer and the third leading cause of cancer-related mortality globally (1). In China, liver cancer ranks as the fourth most prevalent cancer type and the second leading cause of malignant death (2). Hepatocellular carcinoma (HCC), which accounts for approximately 75%–85% of all liver cancers, dominates the incidence and mortality rates among liver malignancies. However, unlike other cancers, cytotoxic chemotherapies have not significantly improved survival outcomes for HCC patients. Immunotherapy has demonstrated superior progression-free survival and overall survival outcomes compared to chemotherapy and tyrosine kinase inhibitors (TKIs) in patients with hepatocellular carcinoma (3, 4). Consequently, therapies involving immune checkpoint inhibitors (ICIs) targeting programmed death-1 (PD-1), programmed death ligand-1 (PD-L1), or cytotoxic T-lymphocyte antigen 4 (CTLA-4) have been approved by the U.S. Food and Drug Administration (FDA) and the National Medical Products Administration of China (NMPA) for use in HCC, either as monotherapy or in combination. However, the current effectiveness of immunotherapy remains unsatisfactory across all HCC patients. Moreover, even when an initial treatment response is achieved, a subset of HCC patients still develops early resistance to immune checkpoint inhibitors. Therefore, identifying effective predictive biomarkers for hepatocellular carcinoma patients receiving immunotherapy could assist clinicians in adopting more effective therapeutic strategies.

Typically, magnesium is found in its ionic form, known as magnesium ion (Mg^2+^). Mg^2+^ plays a pivotal role in numerous cellular functions and biochemical processes, including the transport of potassium and calcium ions, oxidative phosphorylation, synthesis of protein and nucleic acid, modulation of signal transduction, energy metabolism and cell growth (5, 6). Abnormal serum Mg^2+^ levels have been associated with multiple diseases, such as asthma, hypertension, diabetes, atherosclerosis, anxiety, migraine, depression, Parkinson’s disease and Alzheimer’s disease (5, 7). Given magnesium’s critical role in physiological functions, numerous studies have been conducted to investigate the correlation between magnesium and cancers. In cancer patients, magnesium can influence outcomes and overall quality of life. Specifically, in HCC, the serum concentration of Mg decreases at the time of diagnosis and increases after locoregional therapy of the tumor (8). Through magnesium mediated immune synapse formation and specific cytotoxicity, CD8+ T cells are activated (9). It has been previously reported that magnesium ions enhance the activation of effector memory CD8+ T cells via the action of leukocyte function-associated antigen 1 (LFA-1) (10). Furthermore, these findings suggest a potential link between magnesium levels and both prognosis and sensitivity to immunotherapy. A prior study reported that among 1441 patients undergoing immunotherapy, comprising 1042 with lung cancer, 270 with esophageal cancer, and 129 with Hodgkin lymphoma, those with higher serum Mg^2+^ levels exhibited improved progression-free survival and overall survival benefits compared to those with lower serum Mg^2+^ levels (11).

However, its predictive significance regarding progression-free survival and disease control rate in hepatocellular carcinoma patients treated with first-line immunotherapy remains unexplored, and this study represents the first investigation into this association.

## 2 Methods

### 2.1 Patients

This study was an observational, retrospective analysis. Hepatocellular carcinoma patients treated with immune checkpoint inhibitors in a derivation cohort were enrolled at the Department of Medical Oncology, Sir Run Run Shaw Hospital, Zhejiang University between January 2023 and June 2024. The inclusion criteria for patient screening in this study were as follows: (1) patients aged ≥18 years with adequate hematological and organ function; (2) histologically, cytologically, or clinically confirmed locally advanced, or metastatic hepatocellular carcinoma per American Association for the Study of Liver Disease criteria (12); (3) availability of serum magnesium level data within one week prior to the initiation of immunotherapy and every two cycles of treatment until disease progression; (4) immunotherapy administered as the first-line therapy; (5) no prior systemic therapy for hepatocellular carcinoma; (6) measurable disease per the Response Evaluation Criteria in Solid Tumors (RECIST) version 1.1 (13); (7) absence of synchronous or metachronous malignancies; and (8) no use of magnesium supplementation or diuretics agents before the initiation of immunotherapy or during the treatment period.

We formed the independent validation cohort from the Second Department of Medical Oncology, the Fourth Affiliated Hospital of Xinjiang Medical University retrospectively. The inclusion and exclusion criteria were identical to those applied to the derivation cohort.

A formal sample size calculation was not performed as this was a retrospective, population-based study designed to enroll the entire consecutive cohort of patients meeting the inclusion criteria during the specified study period. This approach ensures a comprehensive representation of the target population from two departments.

### 2.2 Data collection

Baseline demographic information and outcome data of each patient were collected retrospectively from electronic medical records. The collected data included patients’ gender, age, comprehensive clinical and pathological characteristics, treatment protocols, date of diagnosis, date of immunotherapy initiation and termination, date of disease progression, date of follow-up or death and so on. The serum Mg^2+^ levels were assayed using the clinical chemistry analyzer (Beckman Coulter, California, USA) following the manufacturer’s protocol. Immunotherapy regimens were selected at the discretion of the treating oncologist; Atezolizumab, Sintilimab, Camrelizumab and Tislelizumab were given typically every three weeks in accordance with established guidelines and indications. All patients underwent follow-up imaging with abdominal computed tomography (CT) or abdominal magnetic resonance imaging (MRI), as well as chest CT after every two cycles of immunotherapy. When clinically indicated, additional positron emission tomography/computed tomography (PET/CT) scan, whole-body bone scan and cranial MRI were performed.

In this study, the primary endpoint was to evaluate the correlation between the disease control rate (DCR) and serum Mg^2+^ levels prior to the initiation of immunotherapy. Disease control included complete response (CR), partial response (PR) and stable disease (SD), as defined by the RECIST criteria version 1.1.

### 2.3 Statistical analyses

The optimal cut-off value for baseline serum Mg^2+^ levels in relation to disease control was determined using receiver operating characteristic (ROC) curve analysis. Patients were then stratified into either the high magnesium (≥the cut-off value) or the low magnesium (<the cut-off value) groups accordingly. Additionally, patients were categorized into four quartile-based groups (Q1, Q2, Q3, Q4) according to the 25th, 50th, and 75th percentiles of pretreatment serum magnesium levels. Specifically, Q1 included values below the 25th percentile, Q2 encompassed values between the 25th and 50th percentiles, Q3 represented values between the 50th and 75th percentiles, and Q4 comprised values above the 75th percentile. Categorical baseline variables were compared using chi-square test or Fisher’s test, while continuous baseline variables were analyzed using the t test or Mann-Whitney U test. Differences in outcomes between the low magnesium and high magnesium groups were assessed using the Log rank test. Progression-free survival (PFS) was defined as the time from the initiation of immunotherapy to disease progression or death from any cause, whichever occurred first, and was estimated using the Kaplan–Meier method. The pre-specified cutoff value determined from the derivation cohort’s ROC analysis, was applied to this validation cohort without modification. The same endpoints were assessed. Univariate and multivariate Cox proportional hazards models were utilized to evaluate the impact of serum Mg^2+^ levels and other clinical variables—including sex, age, presence of macrovascular invasion or extrahepatic metastasis, hepatitis B virus infection status, α-fetoprotein concentration, renal function(estimated Glomerular Filtration Rate), nutritional status, liver function (Albumin-Bilirubin Score), serum albumin, C-reactive protein, type of immunotherapy (monotherapy *vs*. combination therapy), and administration of anti–PD-1, anti–PD-L1, or anti–CTLA-4 antibodies—on PFS. Hazard ratios (HRs) and 95% confidence intervals (CIs) were calculated. All statistical analyses were performed using SPSS software (version 23.0), and a P-value <0.05 was considered statistically significant.

This study was conducted in accordance with the Declaration of Helsinki and was approved by the Ethics Committee of Sir Run Run Shaw Hospital, Zhejiang University.

## 3 Results

### 3.1 Characteristics of the population in derivation cohort

A total of 110 patients with hepatocellular carcinoma who received immune checkpoint inhibitors (ICIs) were enrolled in this derivation cohort. The baseline clinical characteristics of the patients are summarized in **Table 1**. The median age at the initiation of immunotherapy was 62 years, ranging from 31 to 80 years. Among the participants, 97 (88.18%) were male and 13 (11.82%) were female. A total of 58 patients (52.73%) were diagnosed with HCC accompanied by macrovascular invasion, while 52 patients (47.27%) presented with extrahepatic metastasis. Of these patients, 92 cases (83.64%) received immunotherapy in combination with vascular endothelial growth factor (VEGF) inhibitors, and 18 cases (16.36%) underwent immunotherapy as monotherapy. Regarding specific immunotherapy regimens, 92 patients (85.45%) were treated with anti PD-1 antibody, 13 patients (11.82%) with anti PD-L1 antibody and 3 patients (2.73%) with anti CTLA-4 antibody.

**Table 1:** The characteristics of patients in the derivation cohort (n=110).

Baseline Characteristics	n (%)
Age	
Median (range)	62 (31–80)
Gender	
Male	97 (88.18%)
Female	13 (11.82%)
Presence of macrovascular invasion or extrahepatic metastasis	
Macrovascular invasion	58 (52.73%)
Extrahepatic metastasis	52 (47.27%)
Hepatitis B virus infection or not	
With hepatitis B virus infection	95 (86.36%)
Without hepatitis B virus infection	15 (13.64%)
α-fetoprotein concentration	
<400 ng/mL	78 (70.91%)
>400 ng/mL	32 (29.09%)
Immunotherapy monotherapy or in combination	
Monotherapy	18 (16.36%)
In combination	92 (83.64%)
Received anti PD-1 antibody, anti PD-L1 antibody or anti CTLA-4 antibody	
anti PD-1 antibody	94 (85.45%)
anti PD-L1 antibody	13 (11.82%)
anti CTLA-4 antibody	3 (2.73%)
Renal function	
eGFR<60 ml/min	32 (29.09%)
eGFR≥60 ml/min	78 (70.91%)
Nutritional status	
Nutritional Risk Screening 2002 (NRS2002): score of 1	79 (71.82%)
Nutritional Risk Screening 2002 (NRS2002): score of 2	31 (28.18%)
Liver function	
ALBI grade 1	93 (84.55%)
ALBI grade 2	17 (15.45%)
Serum albumin	
<30 g/L	9 (8.18%)
≥30 g/L	101 (91.82%)
C-reactive protein	
<10 mg/L	71 (64.55%)
≥10 mg/L	39 (35.45%)

### 3.2 Survival analyses according to serum Mg^2+^ in derivation cohort

**Figure 1:**
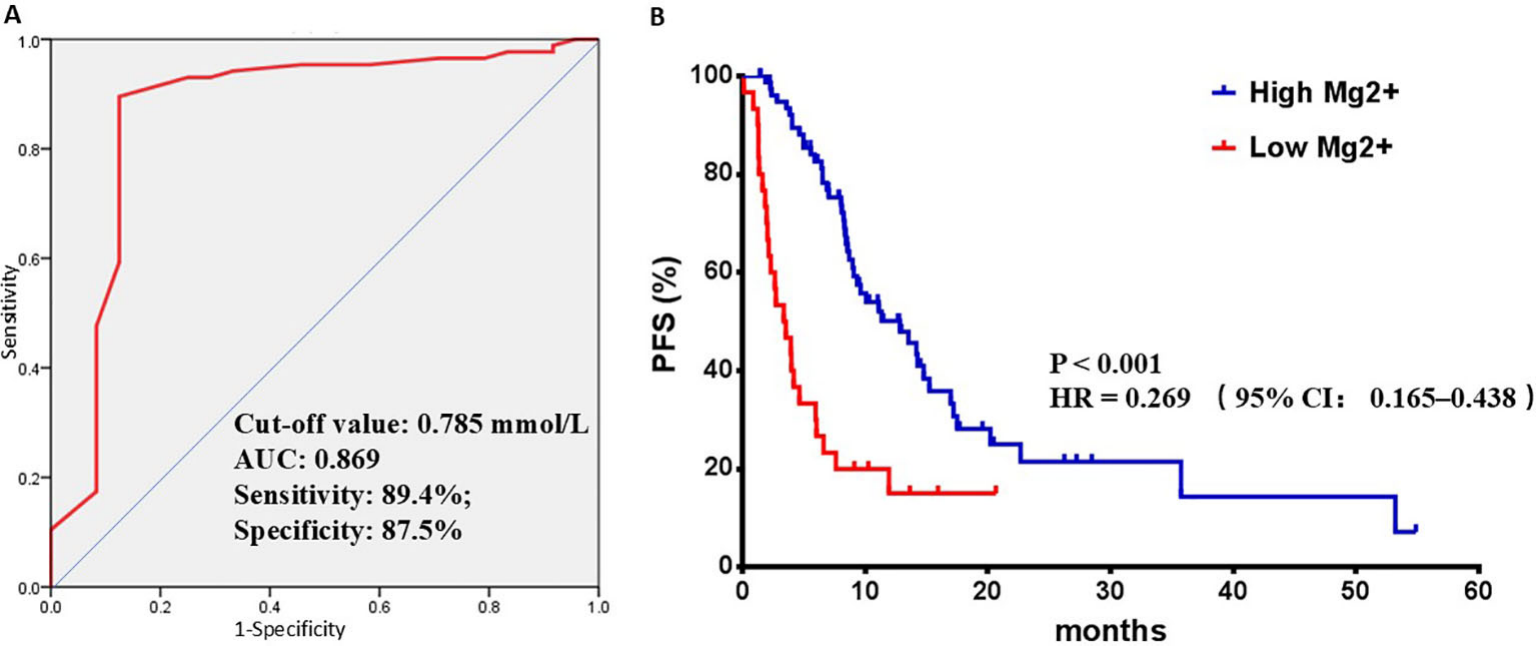
Outcomes of receiver operating characteristic (ROC) curve analyses and survival analyses. **(A)** ROC curve analysis for serum magnesium. **(B)** PFS analysis of high-Mg2+ group and low-Mg2+ group in all eligible patients.

The median pretreatment serum Mg^2+^ level was 0.84 mmol/L, and the median follow-up duration was 18.63 months. The disease control rate was 78.18% (86 out of 110 patients). Partial response (PR) was observed in 14 patients (12.73%), and stable disease (SD) was observed in 72 patients (65.45%). Serum magnesium levels prior to immunotherapy were significantly higher in the disease control group (CR + PR + SD) than compared to progressive disease (PD) group (P < 0.001). In patients in the disease control group, serum magnesium levels remained stable from baseline to progression (P = 0.607). In contrast, patients in the progressive disease group exhibited a significant decrease in serum magnesium levels at the time of progression compared to their baseline (P = 0.038). Based on ROC curve analysis, the optimal cut-off value for pretreatment serum Mg^2+^ was determined to be 0.785 mmol/L, with an area under the curve (AUC) of 0.869 (sensitivity: 89.4%; specificity: 87.5%, **Figure 1A**). Accordingly, patients were categorized into a high-Mg^2+^ group (≥0.785 mmol/L, n = 80) and a low-Mg^2+^ group (<0.785 mmol/L, n = 30). In the low-Mg^2+^ group, the disease control rate was 30% (9 out of 30 patients), whereas in the high-Mg^2+^ group, it was 96.25% (77 out of 80 patients). During follow-up, 71 out of 110 patients (64.55%) experienced disease progression. The baseline characteristics of HCC patients in the high-Mg^2+^ and low-Mg^2+^ groups are summarized in **Table 2**. No significant differences were observed in age, sex, metastatic status, HBV infection status, α-fetoprotein concentration, use of immunotherapy, renal function, nutritional status or liver function. But patients with high magnesium levels had a significantly higher proportion of individuals with serum albumin ≥30 g/L and C-reactive protein <10 mg/L compared to the low magnesium group. The median PFS was significantly longer in the high-Mg^2+^ group compared to the low-Mg^2+^ group (12.83 months *vs*. 3.45 months, P < 0.001, HR 0.269, 95% CI 0.165–0.438, **Figure 1B**).

**Table 2:** The characteristics of patients classified by serum Mg2+ levels in the derivation cohort (n=110).

Baseline characteristics	high-Mg^2+^ group (n=80)	low-Mg^2+^ group (n=30)	P value
Age			
Median (range)	63 (31–80)	63 (46–78)	0.721
Gender			
Male	69 (86.25%)	28 (93.33%)	0.164
Female	11 (13.75%)	2 (6.67%)	
Presence of macrovascular invasion or extrahepatic metastasis			
Macrovascular invasion	46 (57.5%)	12 (40%)	0.063
Extrahepatic metastasis	34 (42.5%)	18 (60%)	
Hepatitis B virus infection or not			
With hepatitis B virus infection	71 (88.75%)	24 (80%)	0.212
Without hepatitis B virus infection	9 (11.25%)	6 (20%)	
α-fetoprotein concentration			
<400 ng/mL	60 (75%)	18 (60%)	0.061
>400 ng/mL	20 (25%)	12 (40%)	
Immunotherapy monotherapy or in combination			
Monotherapy	13 (16.25%)	5 (16.67%)	0.956
In combination	67 (83.75%)	25 (83.33%)	
Received anti PD-1 antibody, anti PD-L1 antibody or anti CTLA-4 antibody			
anti PD-1 antibody	67 (83.75%)	27 (90%)	0.348
anti PD-L1 antibody	11 (13.75%)	2 (6.67%)	
anti CTLA-4 antibody	2 (2.5%)	1 (3.33%)	
Renal function			
eGFR<60 ml/min	23	9	0.898
eGFR≥60 ml/min	57	21	
Nutritional status			
Nutritional Risk Screening 2002 (NRS2002): score of 1	60	19	0.226
Nutritional Risk Screening 2002 (NRS2002): score of 2	20	11	
Liver function			
ALBI grade 1	66	27	0.395
ALBI grade 2	14	3	
Serum albumin			
<30 g/L	4	5	0.047
≥30 g/L	76	25	
C-reactive protein			
<10 mg/L	57	14	0.016
≥10 mg/L	23	16	

**Figure 2:**
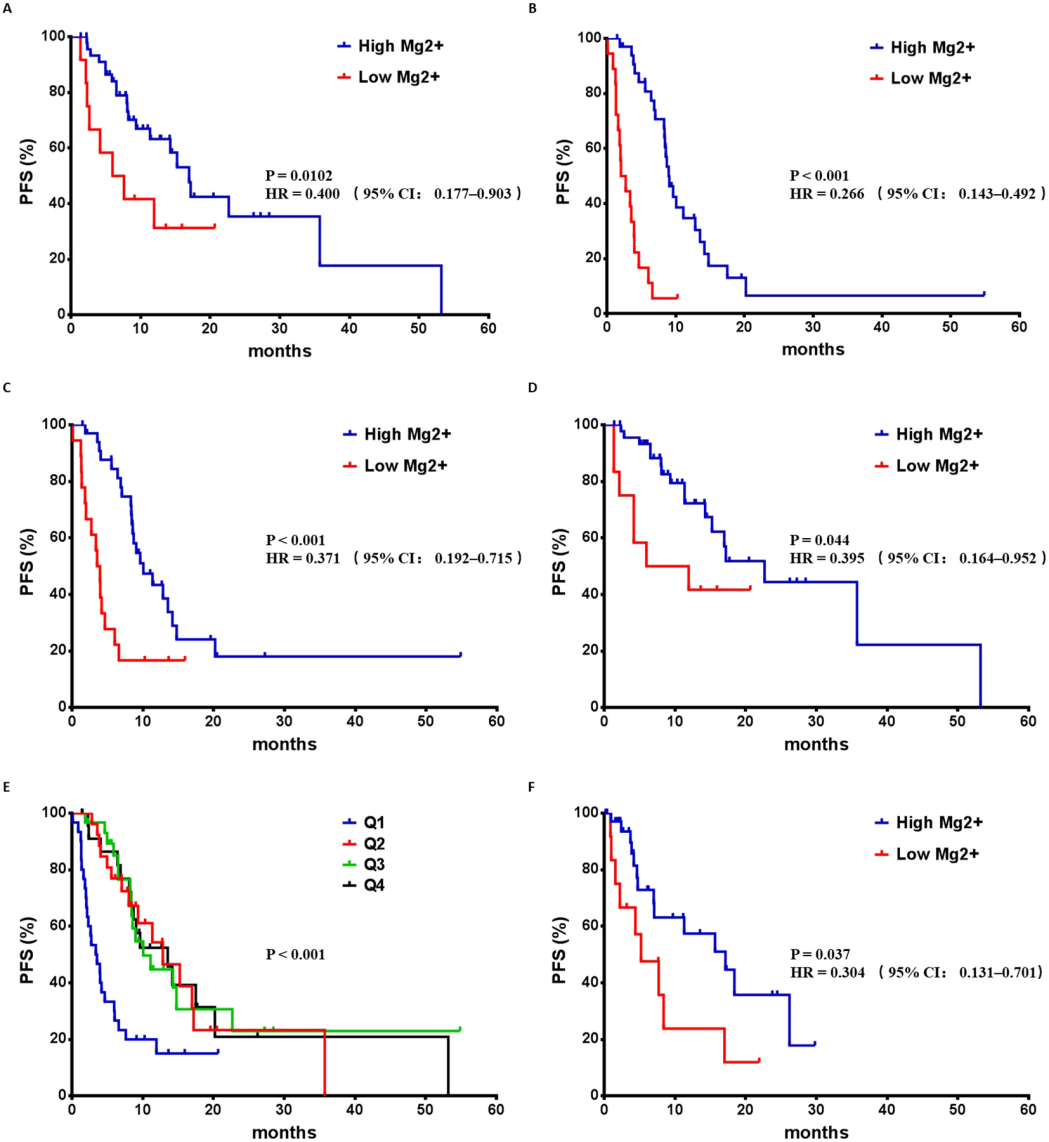
Kaplan–Meier survival curves of progression-free survival in subgroup analyses. **(A)** PFS analysis of HCC patients with macrovascular invasion. **(B)** PFS analysis of HCC patients with extrahepatic metastasis. **(C)** PFS analysis of HCC patients without macrovascular invasion. **(D)** PFS analysis of HCC patients without extrahepatic metastasis. **(E)** PFS across the quartiles. **(F)** PFS analysis of high-Mg2+ group and low-Mg2+ group in validation cohort.

In subgroup analyses, among patients with macrovascular invasion, the median PFS in high-Mg^2+^ group was significantly longer than that in low-Mg^2+^ group (16.97 months *vs*. 6.78 months, P = 0.0102, HR 0.400, 95% CI 0.177–0.903, **Figure 2A**). For patients with extrahepatic metastasis, the median PFS in the high-Mg^2+^ group was 8.97 months, while that in the low-Mg^2+^ group was 2.38 months (P < 0.001, HR 0.266, 95% CI 0.143–0.492, **Figure 2B**). Similarly, in patients without macrovascular invasion, a significant PFS benefit was observed in the high-Mg^2+^ group (10.07 months *vs*. 3.73 months, P < 0.001, HR 0.371, 95% CI 0.192–0.715) (**Figure 2C**). This association was also evident in patients without extrahepatic metastases, where the high-Mg^2+^ group had a median PFS of 22.67 months compared to 8.95 months in the low-Mg^2+^ group (P = 0.044, HR 0.395, 95% CI 0.164–0.952) (**Figure 2D**).

Kaplan-Meier analysis demonstrated a significant and graded improvement in PFS across the quartiles (P < 0.001, **Figure 2E**). The median PFS for Q1, Q2, Q3, and Q4 was 3.37, 12.83, 10.07, and 13.53 months, respectively.

Univariate and multivariate analyses were conducted based on the patients’ baseline characteristics (**Table 3**). The results indicated that baseline elevated serum Mg^2+^ levels (≥0.785 mmol/L) and the presence of macrovascular invasion, rather than extrahepatic metastasis, were independent predictive factors associated with prolonged PFS in HCC patients. However, C-reactive protein, which was significant in univariate analysis, lost its independent significance in the multivariate model.

**Table 3:** Univariate and multivariate analyses for the response to immunotherapy in the derivation cohort.

Variables	Univariate analyses			Multivariate analyses		
	HR	95% CI	P value	HR	95% CI	P value
Sex: Female	0.620	0.128-3.009	0.553			
Age: <62	0.729	0.281-1.887	0.514			
HBV status: with HBV	0.733	0.211-2.550	0.626			
Metastatic status: extrahepatic metastasis	3.539	1.329-9.426	0.011	2.557	1.559-4.196	<0.001
AFP: >400	2.078	0.808-5.347	0.129			
Renal function: eGFR<60	0.934	0.555-1.572	0.797			
Nutritional status:NRS2002 score of 1	0.784	0.469-1.310	0.352			
Liver function: ALBI grade 1	1.074	0.531-2.249	0.850			
Serum albumin:<30	1.813	0.776-4.235	0.169			
C-reactive protein: <10	0.587	0.362-0.952	0.031	0.625	0.379-1.030	0.065
ICIs mono./combination: combination	0.677	0.215-2.134	0.505			
Agent types (anti-PD-L1/anti-PD-1/anti-CTLA-4: anti PD-L1	0.705	0.221-2.252	0.555			
Mg^2+^: <0.785	3.135	2.755-10.220	<0.001	3.804	2.258-6.410	<0.001

### 3.3 Characteristics and survival analyses in validation cohort

The clinical utility of the 0.785 mmol/L serum magnesium cutoff was assessed in the independent external validation cohort (n=48). The baseline characteristics of the validation cohort are summarized in **Supplementary Table 1** and were largely comparable to the derivation cohort.

Patients in the validation cohort with pretreatment serum magnesium ≥0.785 mmol/L had a significantly higher disease control rate (91.67% *vs*. 58.33%, P < 0.001) compared to the low-magnesium group. More importantly, Kaplan-Meier analysis confirmed a significant prolongation of median PFS in the high-magnesium group versus the low-magnesium group (17.13 months *vs*. 5.20 months, P = 0.037, HR 0.304, 95% CI 0.131–0.701, **Figure 2F**).

## 4 Discussion

Immunotherapies have demonstrated significant clinical benefits in patients with hepatocellular carcinoma. The IMbrave 150 study revealed that a therapy containing Atezolizumab (an anti-PD-L1 antibody) improved progression-free survival and overall survival outcomes in patients with advanced HCC (3). Similarly, the ORIENT-32 study conducted in the Chinese population showed that a therapy containing Sintilimab (an anti-PD-1 antibody) resulted in superior clinical outcomes (4). The success of Atezolizumab and Sintilimab in HCC heralds a new era of systemic treatment for this disease. However, the disease control rate of immunotherapy was 73.6% in the IMbrave 150 study and 72% in the ORIENT-32 study. It is well established that hepatocellular carcinoma is a highly heterogeneous disease, as evidenced by the fact that nearly a quarter of HCC patients do not respond to ICIs. Additionally, a subset of advanced HCC patients fails to achieve long-term benefit from immunotherapy due to acquired drug resistance. Therefore, further investigation is warranted to elucidate the mechanisms underlying the variable responses to ICIs in HCC patients and to identify strategies to overcome resistance.

Our study represents the first retrospective investigation evaluating the association between serum magnesium levels and clinical outcomes in HCC patients treated with ICIs. Our findings indicate that higher pretreatment serum magnesium levels are correlated with improved clinical outcomes, including an increased disease control rate (CR + PR + SD) and prolonged PFS in HCC patients undergoing ICI therapy. In our statistical analyses, neither the treatment regimen (monotherapy *vs*. combination) nor the agent type (anti-PD-1 *vs*. anti-PD-L1) was significantly associated with PFS in univariate analysis. This suggests that the predictive value of pretreatment serum magnesium may be consistent across these specific variations in immunotherapy within our cohort. Even after controlling for renal function, nutritional status, liver function (ALBI grade), and systemic inflammation (C-reactive protein), serum magnesium remained an independent predictor of PFS. The most significant strength of our study is the successful external validation in an independent cohort from a different department. The fact that the cutoff of 0.785 mmol/L consistently stratified patients in both the derivation and validation cohorts markedly reduces the risk of bias. This successful external validation is a critical step, significantly enhancing the potential generalizability of serum magnesium as a clinically useful predictive tool. These findings are consistent with results from prior studies conducted in other common malignancies. An investigation revealed that non-small cell lung cancer patients treated with Durvalumab (an anti PD-L1 antibody) who experienced hypomagnesemia had decreased overall survival compared to those with normomagnesemia (10). Furthermore, a study indicated that patients with lung cancer, esophageal cancer, and Hodgkin lymphoma who had high serum magnesium levels (≥0.79 mmol/L) showed favorable responses to ICI treatment, along with longer progression-free survival and overall survival (11). Notably, patients in the high magnesium group also exhibited a higher overall response rate, including complete response and partial response, compared to those in the low magnesium group (11). The novelty of our work does not stem from proposing a new biological mechanism, but in the successful translation and validation of an established concept within a specific clinical context. This finding positions serum magnesium as a practical and readily accessible biomarker, potentially paving the way for future investigations. An important question raised by our findings is whether this association is specific to the immunotherapy or represents a broader predictive indicator applicable to non-immunotherapy regimens, such as tyrosine kinase inhibitors or chemotherapy. For example, a non-HCC cancer type, Xu et al. demonstrated that non-small cell lung cancer patients with higher pre-treatment Mg^2+^ levels exhibited longer progression-free survival and overall survival when receiving epidermal growth factor receptor TKIs therapy (14). This finding indicates that magnesium may modulate anti-tumor responses in other cancer contexts. However, ICI-based therapies have become the standard first-line treatment for eligible patients with HCC. A control group of comparable patients receiving non-immunotherapy treatments is scarce and likely subject to substantial selection bias, as these individuals often have contraindications to immunotherapy. Therefore, the most urgent clinical question has evolved: identifying biomarkers capable of stratifying outcomes among patients who are expected to receive immunotherapy is crucial. Future prospective studies that include baseline magnesium assessment in all HCC patients, combined with comprehensive biomarker profiling, could further clarify its independent prognostic and predictive value.

In recent years, advances in genome sequencing—such as single-cell RNA sequencing—as well as immune cell identification strategies and microbiota analysis have generated significant potential for identifying novel prognostic biomarkers. These biomarkers could enable more accurate selection of patients most likely to benefit from immune checkpoint inhibitors. However, many of these techniques are costly and time-consuming, limiting their large-scale application in real-world settings. In contrast, serum magnesium is a routine biochemical electrolyte test with high clinical accessibility. It is more cost-effective, provides rapid results, and allows for repeated testing and dynamic monitoring, making it better suited for widespread use in practical clinical scenarios. In previous studies, the neutrophil-to-lymphocyte ratio and platelet-to-lymphocyte ratio (NLR and PLR) have been established as inflammatory cell markers and potential predictive biomarkers in HCC patients undergoing immunotherapy (15). A decrease in NLR has been associated with improved survival outcomes; however, no significant association between NLR and ORR has been observed (16). This inconsistency in the relationship with treatment response raises concerns regarding the reliability of NLR and PLR as predictive biomarkers for guiding the use of immune checkpoint inhibitors (ICIs) in patients with HCC.

Magnesium is indispensable for maintaining health and sustaining life. Mg^2+^ plays a critical role in both innate and acquired immune responses and is involved in the regulation of DNA repair mechanisms (17). Magnesium deficiency contributes to immune dysfunction and can induce DNA mutations, potentially leading to tumorigenesis. To directly assess the impact of systemic magnesium levels on the antigen-specific cytotoxic activity of memory CD8+ T cells, *in vivo* killing assays were conducted. Antigen-specific target cell clearance was reduced in Mg^2+^-deficient hosts, and cytotoxicity was partially restored by co-injection of magnesium with target cells. In this model, dietary Mg^2+^ depletion also impaired the effector function of memory CD8+ T cells, whereas supplementation of the bacterial inoculum with Mg^2+^ significantly enhanced bacterial clearance (10). Intratumoral delivery of Mg^2+^ can enhance memory CD8+ T cell-mediated anti-tumor immunity. By increasing LFA-1 outside-in signaling, extracellular Mg^2+^ activates memory CD8+ T cells during immune surveillance in tissues with high versus low Mg^2+^ levels (10). The magnesium transporter 1 (MAGT1) is a critical regulator of basal intracellular magnesium levels. Individuals with genetic deficiencies in MAGT1 exhibit a significantly increased predisposition to lymphoma. Previous studies have shown that reduced intracellular magnesium levels lead to impaired expression of the natural killer cell activating receptor in both natural killer (NK) and CD8+ T cells, thereby compromising cytolytic responses (18). Magnesium modulates the immune system through the activation of STING pathway (stimulator of IFN genes) and the induction of type I IFN expression, promoting dendritic cell maturation and antigen presentation, thereby enhancing the antitumor effect of T cells. Research has shown that the mitogen-activated protein kinase (MAPK) signaling pathway, which is regulated by magnesium, is activated in over 50% of human hepatocellular carcinoma cases (19). Enhancing the immune response can improve the therapeutic efficacy of immune checkpoint inhibitors. The reduced efficacy of immune therapy in liver cancer patients may be attributed to the high magnesium affinity exhibited by tumors of greater malignancy, which leads to hypomagnesemia. Cellular magnesium homeostasis is primarily maintained by TRPM7, whose overexpression and/or activation has been implicated in the growth and proliferation of hepatocellular carcinoma (8). Mg^2+^ deficiency has been linked to inflammation and elevated levels of free radicals, both of which can contribute to oxidative DNA damage and subsequent tumor development. Reduced Mg^2+^ concentrations in serum and liver tissue may promote disease progression by impairing mitochondrial function, disrupting protein kinase C translocation, and exacerbating inflammatory responses, oxidative stress, and metabolic dysregulation. A retrospective study demonstrated that higher serum Mg^2+^ levels are significantly associated with a lower risk of HCC in patients with nonalcoholic fatty liver disease (20). Another retrospective analysis indicated that serum Mg^2+^ levels were markedly lower in cirrhotic patients with HCC compared to those without HCC (8). Our results, which showed lower serum magnesium levels were associated with decreased clinical outcomes and further declined with disease progression, support the hypothesis that HCC may have an increased demand for magnesium to sustain cell proliferation, thereby leading to reduced serum magnesium concentrations.

It has been previously reported that feeding mice a magnesium-deficient diet accelerates the genetic instability, as well as the invasion and metastasis of cancer cells (21). Previous studies have indicated that higher dietary or drinking water intake of magnesium is associated with a decreased risk of colorectal cancer, liver cancer and lung cancer (22, 23). Our study provides evidence that a significant decrease in serum magnesium occurs specifically at the time of disease progression among patients who had high baseline levels but fail to achieve disease control, whereas levels remain stable in those who respond with disease control. This indicates that the dynamic decline in magnesium, rather than baseline concentration alone, may represent a biological event associated with the development of treatment resistance. These findings underscore the importance of a magnesium-enriched diet in enhancing the efficacy of immunotherapy. However, several epidemiological studies have revealed that individuals often consume dietary magnesium below the Recommended Daily Allowance (RDA) of 320 to 420 mg/day (24). Oral magnesium supplementation is more commonly utilized due to its ability to consistently elevate serum magnesium levels. In both *in vitro* and *in vivo* testing, organic magnesium compounds, such as magnesium citrate, demonstrate superior bioavailability compared to inorganic magnesium compounds (24). Furthermore, oral magnesium supplementation is generally well tolerated, with diarrhea, if present, being mild. Additionally, Wu et al. found that oral administration of magnesium-L-threonate enhances analgesia while reducing the required dosage of opioids, thereby improving the quality of life in advanced cancer patients (25). There is robust evidence demonstrating the involvement of magnesium ions in modulating immune responses; however, further mechanistic investigations and prospective studies are necessary to validate these findings. As a critical regulatory “switch” in tumor growth, magnesium ions represent a promising therapeutic target for cancer and hold significant potential for broad clinical applications. In the future, multicenter prospective clinical trials could be designed to investigate whether oral magnesium supplementation, when combined with immunotherapy, can enhance the therapeutic efficacy of immunotherapy in hepatocellular carcinoma.

There are several limitations to our study. First, our retrospective study was conducted with a limited sample size, which may introduce bias. Second, our cohort was highly male-predominant. However, this sex imbalance reflects the well-documented epidemiology of HCC, which has a strong male predominance globally. Prior research has demonstrated that serum magnesium levels are generally higher in healthy males compared to females (26). The sex ratio limits our ability to robustly investigate the relationship between magnesium and immunotherapy responses in female patients. Moreover, our study enrolled only Chinese hepatocellular carcinoma patients, and differences between Eastern and Western populations may exist. Our cohort was predominantly composed of patients with hepatitis B virus related HCC, reflecting the high prevalence of HBV as an etiology in Eastern Asian populations. In contrast, the epidemiological landscape of HCC in Western populations is characterized by a higher prevalence of alcohol-related liver disease and non-alcoholic steatohepatitis (NASH) (27). It has been suggested that the underlying tumor immune microenvironment may differ between HCCs of viral and non-viral origins. For instance, NASH-related HCC is often associated with aberrant T cell activation and potentially more immunosuppressive microenvironment, which might be less responsive to immunotherapy (28). Considering these limitations, further multicenter research involving larger and more diverse sample sizes is necessary to provide compelling evidence.

## 5 Conclusion

In conclusion, our findings demonstrate that pretreatment serum magnesium levels serve as a potential independent predictive biomarker for immunotherapy response in patients with hepatocellular carcinoma receiving first-line immune checkpoint inhibitors. These results underscore the need for future prospective studies in large, homogeneously treated cohorts to definitively validate the association between baseline serum magnesium levels and overall survival, and to establish its clinical utility as a reliable prognostic marker.

## Data Availability

The raw data supporting the conclusions of this article will be made available by the authors, without undue reservation.
