# Supplementary material for: Serum magnesium predictive value in hepatocellular carcinoma patients on first-line immunotherapy: a retrospective study
Source: Front Immunol. 2026 Jan 5;16:1732557. doi: 10.3389/fimmu.2025.1732557 (PMC12813027; doi:10.3389/fimmu.2025.1732557)
Supplement: Supplementary file 1 [file Table1.docx]

Supplementary Table1 The characteristics of patients in the validation cohort (n=48).

| Baseline Characteristics | n (%) |
| --- | --- |
| Age |  |
| Median (range) | 60 (40–83) |
| Gender |  |
| Male | 41 (85.42%) |
| Female | 7 (14.58%) |
| Presence of macrovascular  invasion or extrahepatic  metastasis |  |
| Macrovascular invasion | 32 (66.67%) |
| Extrahepatic metastasis | 25 (52.08%) |
| Hepatitis B virus infection or not |  |
| With hepatitis B virus infection | 32 (66.67%) |
| Without hepatitis B virus infection | 16 (33.33%) |
| α-fetoprotein concentration |  |
| ＜400 ng/mL | 35 (72.92%) |
| ＞400 ng/mL | 13 (27.08%) |
| Immunotherapy monotherapy or in combination |  |
| Monotherapy | 4 (8.33%) |
| In combination | 44 (91.67%) |
| Received anti PD-1 antibody, anti PD-L1 antibody or anti CTLA-4 antibody |  |
| anti PD-1 antibody | 48 (100.00%) |
| Renal function |  |
| eGFR<60 ml/min | 13 (27.08%) |
| eGFR≥60 ml/min | 35 (72.92%) |
| Nutritional status |  |
| Nutritional Risk Screening 2002 (NRS2002): score of 1 | 39 (81.25%) |
| Nutritional Risk Screening 2002 (NRS2002): score of 2 | 9 (18.75%) |
| Liver function |  |
| ALBI grade 1‌ | 38 (79.17%) |
| ALBI grade 2 | 10 (20.83%) |
| Serum albumin |  |
| <30 g/L | 7 (14.58%) |
| ≥30 g/L | 41 (85.42%) |
| C-reactive protein |  |
| <10 mg/L | 36 (75%) |
| ≥10 mg/L | 12 (25%) |
